# Supplementary material for: Modulatory effects of rutin and vitamin A on hyperglycemia induced glycation, oxidative stress and inflammation in high-fat-fructose diet animal model
Source: PLoS One. 2024 May 9;19(5):e0303060. doi: 10.1371/journal.pone.0303060 (PMC11081234; doi:10.1371/journal.pone.0303060)
Supplement: S1 Fig — (DOCX) [file pone.0303060.s001.docx]

**Supplementary Information for**

**Modulatory effects of Rutin and Vitamin A Supplementation on Hyperglycemia induced Glycation, Oxidative stress and Inflammation in High-Fat-Fructose Diet Animal Model**

### **Oral glucose tolerance test (OGTT)**

### After a 12-hour fast at the conclusion of the 42 days experiment, rats were put through an oral glucose tolerance test before being dissected. A 2.5g/kg D-glucose solution was given orally an hour after the treatment. Each rat had blood drawn from a vein in its tail before receiving glucose as well as 30, 60-, 90-, 120-, and 180-minutes interval to measure blood glucose levels. Oral Glucose Tolerance Test (OGTT) was performed on the 41^st^ day of treatments. After 1 hr. of administering various treatments to relative groups, glucose (2g/kg) was administration and blood glucose levels were measured at 0, 30, 60, 90, 120 and 180 minutes. At 30 minutes, the HFFD group's blood glucose levels peaked (391.4 ± 12.8 mg/dL). The maximal blood glucose concentration was seen at 30 minutes in the metformin (173 ± 2.8 mg/dL), rutin (205.8 ± 6.6 mg/dL), vitamin A (195 ± 5.3 mg/dL) and combination treatment (197 ± 4.2 mg/dL) groups. Compared to the HFFD group shown in Fig. 1, a considerable drop in blood glucose levels was seen after 30 minutes with combination treatment.

##### Fig 1. Oral Glucose Tolerance assessment in Normal Control, HFFD Metformin (120mg/kg), Rutin (100mg/kg), Vitamin A (43IU/kg), Rutin (100mg/kg) + Vitamin A (43IU/kg)

Each value represents Mean ± SEM (n=5). # (when compared with normal control) and * (when compared with HFFD) presents significant differences (p < 0.05) among treatments was observed after analysis by one way ANOVA and multiple comparison followed by Dunnett’s post-hoc test.
