# Supplementary material for: Modulatory effects of rutin and vitamin A on hyperglycemia induced glycation, oxidative stress and inflammation in high-fat-fructose diet animal model
Source: PLoS One. 2024 May 9;19(5):e0303060. doi: 10.1371/journal.pone.0303060 (PMC11081234; doi:10.1371/journal.pone.0303060)
Supplement: S1 Table — (DOCX) [file pone.0303060.s002.docx]

**Supplementary Information for**

**Modulatory effects of Rutin and Vitamin A Supplementation on Hyperglycemia induced Glycation, Oxidative stress and Inflammation in High-Fat-Fructose Diet Animal Model**

**Molecular docking study of Rutin**

**Table I:** ***In-silico* studies expressing binding affinity (kcal/mol), hydrogen binding, hydrophobic and electrostatic interactions with distances in Angstrom for investigated ligand Rutin with target proteins.**

| **Rutin Ligand &**  **Binding Affinity, ΔG (kcal/mol)** | **Residue Distance (Å)** | **Residue Pocket Amino Acids** | **Type of Interactions** | **Rutin Ligand &**  **Binding Affinity, ΔG (kcal/mol)** | **Residue Distance (Å)** | **Residue Pocket Amino Acids** | **Type of Interactions** |
| --- | --- | --- | --- | --- | --- | --- | --- |
| 1A3Q  (NF-κB)  -7.7 | 2.84  2.54  2.44  2.67  3.32  4.35  3.93  4.95  4.81  4.95  4.17 | ARG103  GLN157 -  GLU92  ASP94  ARG103  ARG103  ARG193  ARG160  SER161  ALA104  ARG160 | H-Bond  H-Bond  H-Bond  H-Bond  Carbon H-Bond  Pi-Cation  Pi-Cation  Pi-Cation  Pi-Alkyl  Pi-Alkyl  Pi-Alkyl | 1DGB (Catalase)  -8.9 | 1.90  3.14  4.86  3.8  3.77  4.10  4.48  4.65  5.42 | H-O bond  C-H bond  HIS466  HIS466  VAL126  ARG127  ARG127  ARG127  VAL182 | H-Bond  Pi-Donor H-Bond  Pi-Pi Stacked  Pi-Pi Stacked  Alkyl  Pi-Alkyl  Pi-Alkyl  Pi-Alkyl  Pi-Alkyl |
| 1HZ2 (MDA)  -7.6 | 2.25  2.48  2.00  2.50  2.18  2.47  2.38  3.38 | DT8  DG10  DG11:O3'  DC12:OP1  H-O bond  H-N bond  H-O bond  DG11 | H-Bond  H-Bond  H-Bond  H-Bond  H-Bond  H-Bond  H-Bond  Carbon H-Bond | 2L3Y (IL-6)  -7.9 | 2.49  2.14  2.45  1.81  1.92  2.37  4.01  4.66  4.61 | ASN75  LEU90  LYS91  H-O bond  GLY77  LEU90  ARG74  ARG74  LEU89 | H-Bond  H-Bond  H-Bond  H-Bond  H-Bond  H-Bond  Pi-Alkyl  Pi-Alkyl  Pi-Alkyl |
| 2NZT (hexokinase 2)  -9.3 | 2.61  2.72  2.20  2.13  2.57  3.25  3.14  3.48  3.39  3.50  4.48  5.34  4.44 | SER70  PHE768  ASP814  H-O bond  ASP814  SER70  ARG69  VAL459  LEU463  LEU463  ARG69  LEU163  ARG69 | H-Bond  H-Bond  H-Bond  H-Bond  H-Bond  H-Bond  Pi-Donor H-Bond  Pi-Sigma  Pi-Sigma  Pi-Sigma  Pi-Alkyl  Pi-Alkyl  Pi-Alkyl | 2P31 (GPx)  -8.0 | 2.81  2.24  2.62  2.46  1.91  2.23  2.36  3.70  3.29  3.30  4.13  4.20 | LYS42  LYS42  LYS42  TYR43  PHE79  H-O bond  MET114  HIS78  GLY75  ASN80  PRO113  PRO113 | H-Bond  H-Bond  H-Bond  H-Bond  H-Bond  H-Bond  H-Bond  H-Bond  H-Bond  H-Bond  Pi-Alkyl  Pi-Alkyl |
| 5D14  (IL-8)  -6.3 | 1.98  2.88  2.31  2.26  2.05  2.17  2.13  3.37  3.95 | VAL25  LEU23  H-O bond  H-O bond  LEU3  LYS1  GLU27  HIS31  ILE26 | H-Bond  H-Bond  H-Bond  H-Bond  H-Bond  H-Bond  H-Bond  H-Bond  Pi-Sigma | 7WT1 (Glo-1)  -6.6 | 2.77  1.78  2.40  1.7  3.64  3.83  5.70  5.14 | GLY53:O  H-O bond  VAL51  H-O bond  TYR74  TYR74  TYR74  ALA130 | H-Bond  H-Bond  H-Bond  H-Bond  Pi-Pi Stacked  Pi-Pi Stacked  Pi-Pi Stacked  Pi-Alkyl |
