# Supplementary material for: Modulatory effects of rutin and vitamin A on hyperglycemia induced glycation, oxidative stress and inflammation in high-fat-fructose diet animal model
Source: PLoS One. 2024 May 9;19(5):e0303060. doi: 10.1371/journal.pone.0303060 (PMC11081234; doi:10.1371/journal.pone.0303060)
Supplement: S2 Table — (DOCX) [file pone.0303060.s003.docx]

**Supplementary Information for**

**Modulatory effects of Rutin and Vitamin A Supplementation on Hyperglycemia induced Glycation, Oxidative stress and Inflammation in High-Fat-Fructose Diet Animal Model**

**Molecular docking study of Vitamin A**

**Table II:** **Binding affinity (kcal/mol), hydrogen binding, hydrophobic and electrostatic interactions with distances in Angstrom for investigated ligand Vitamin A with target proteins.**

| **Rutin Ligand &**  **Binding Affinity, ΔG (kcal/mol)** | **Residue Distance (Å)** | **Residue Pocket Amino Acids** | **Type of Interactions** | **Rutin Ligand &**  **Binding Affinity, ΔG (kcal/mol)** | **Residue Distance (Å)** | **Residue Pocket Amino Acids** | **Type of Interactions** |
| --- | --- | --- | --- | --- | --- | --- | --- |
| 1A3Q  (NF-κB)  -4.3 | 4.97  5.22  5.16  5.03 | PRO208  LYS210  PRO211  PHE197 | Hydrophobic  Hydrophobic  Hydrophobic  Hydrophobic | 1DGB (Catalase)  -6.1 | 4.06  4.3  5.27  5.07  4.30  4.72  5.17 | ALA289  PRO347  PRO347  TYR231  PHE286  PHE297  TYR425 | Hydrophobic  Hydrophobic  Hydrophobic  Hydrophobic  Hydrophobic  Hydrophobic  Hydrophobic |
| 1HZ2 (MDA)  -5.6 | 2.34 | DA9 | H-Bond | 2L3Y (IL-6)  -5.5 | 3.68  4.44  3.46  5.41  4.83 | GLN85  ILE72  ARG74  ARG74  LEU89 | H-Bond Hydrophobic  Hydrophobic  Hydrophobic  Hydrophobic |
| 2NZT (hexokinase 2)  -5.0 | 2.41  5.04  3.70  5.40  4.76 | GLN799  LEU795  ALA802  LEU867  ILE803 | H-Bond Hydrophobic  Hydrophobic  Hydrophobic  Hydrophobic | 2P31 (GPx)  -5.0 | 2.65  3.688  4.510 | HIS78  TYR43  PRO113 | H-Bond  H-Bond  Alkyl |
| 5D14  (IL-8)  -5.3 | 3.98  4.78  4.14 | PHE19  PHE19  PHE19 | Hydrophobic  Hydrophobic  Hydrophobic | 7WT1 (Glo-1)  -5.3 | 5.04  3.87 | ALA137  TYR74 | Hydrophobic  Hydrophobic |
